# Role of Vaginal Microbiota Dysbiosis in Gynecological Diseases and the Potential Interventions

**DOI:** 10.3389/fmicb.2021.643422

**Published:** 2021-06-18

**Authors:** Yiwen Han, Zhaoxia Liu, Tingtao Chen

**Affiliations:** ^1^Department of Obstetrics and Gynecology, The Second Affiliated Hospital of Nanchang University, Nanchang, China; ^2^Queen Mary School, Nanchang University, Nanchang, China; ^3^National Engineering Research Center for Bioengineering Drugs and the Technologies, Institute of Translational Medicine, Nanchang University, Nanchang, China

**Keywords:** vaginal microbiota transplantation, bacterial vaginitis, antibiotics, *Lactobacillus*, gynecological diseases

## Abstract

Vaginal microbiota dysbiosis, characterized by the loss of *Lactobacillus* dominance and increase of microbial diversity, is closely related to gynecological diseases; thus, intervention on microbiota composition is significant and promising in the treatment of gynecological diseases. Currently, antibiotics and/or probiotics are the mainstay of treatment, which show favorable therapeutic effects but also bring problems such as drug resistance and high recurrence. In this review, we discuss the role of vaginal microbiota dysbiosis in various gynecological infectious and non-infectious diseases, as well as the current and potential interventions.

## Introduction

The vagina is an important and complex ecosystem, dominated by *Lactobacillus*, but also containing a small number of fungi and parasites, and the balanced microbial communities are vital for female health (Bradford and Ravel, 2017; Gupta et al., 2019). However, the microbial balance can be disrupted and leads to various infectious diseases, characterized by overgrowth of anaerobic bacteria (agent of bacterial vaginitis and atrophic vaginitis, BV and AV) and *Candida albicans* (agent of vulvovaginal candidiasis, VVC), and infections of *Trichomonas vaginalis* (agent of trichomonal vaginitis), *Neisseria gonorrhoeae* (agent of gonorrhea), *Mycoplasma genitalium* (agent of cervicitis), *Chlamydia trachomatis* (agent of pelvic inflammatory disease, PID), and various viruses including human papillomavirus (HPV, agent of cervical cancer), herpes simplex virus-2 (HSV-2, agent of genital ulcers), and human immunodeficiency virus (HIV, agent of acquired immunodeficiency syndrome, AIDS) (Gupta et al., 2019). In addition, some non-infectious diseases, e.g., induced abortions (with BV microbiome, etc.), intrauterine adhesions (IUA, with reduced *Lactobacillus* and increased *Gardnerella*, *Prevotella*, etc.), miscarriage (with BV microbiome), preterm (with BV microbiome), infertility (with BV microbiome), polycystic ovarian syndrome (PCOS, with reduced *Lactobacillus crispatus*, and increased *mycoplasma* and *Prevotella*), uterine fibroid (with increased *Lactobacillus iners*), and menstrual disorders (with increased uterine *Gardnerella*, *Prevotella*, *Sneathia*, and *Veillonella*), also show associations with microbial dysbiosis (Hay, 2004; Chen et al., 2017; Pelzer et al., 2018; Liu et al., 2019; Hong et al., 2020a, b), posing a serious threat to women’s reproductive health.

At present, the most conventional treatment strategy for microbial disorders is antibiotics (metronidazole, clotrimazole, azithromycin, etc.), which has a good therapy effect while accompanied with various adverse effects and recurrence (Cudmore et al., 2004; Bradshaw et al., 2006; Xie et al., 2017; Khosropour et al., 2018; Paez-Canro et al., 2019). Recently, probiotics based on *Lactobacillus* have shown promise in treating not only infectious diseases (e.g., BV, fungal infection, and urinary tract infections) but also non-infectious diseases (e.g., preterm, infertility, and PCOS) (Hanson et al., 2016; Lopez-Moreno and Aguilera, 2020). However, the treatment outcome of probiotics is usually mixed, which may be due to the fact that these diseases are usually caused by multiple microbes rather than one. Excitingly in 2019, Ahinoam et al. conducted a clinical study of vaginal microbiota transplantation (VMT) in five patients with recurrent BV, finding that four of them achieved long-term remission and established a long-term vaginal microbiota dominated by *Lactobacillus* (Lev-Sagie et al., 2019). Therefore, intervention on vaginal microbiota is significant and promising in treating gynecological diseases. Thus, in this review, we elaborated the role of microbiota dysbiosis in various gynecological infectious and non-infectious diseases, and the current and potential interventions.

## Vagina and Vaginal Microbiota

The vagina is a stretchable, muscular duct connecting the uterus and external genitalia, and is responsible for the physiological functions of female sexual intercourse, menstrual discharge, and delivery of the fetus (Farage and Maibach, 2006). Its mucosal system, consisted of a stratified squamous epithelium and cervicovaginal fluid (CVF), is vital in maintaining vaginal health by immune response, antimicrobial products (e.g., B-defensin), finely balanced microbial communities, etc. (Torcia, 2019). Among these, the vaginal microbiota is the most changeable and vulnerable one in response to internal and external stimulus (Gajer et al., 2012).

Recently, the detailed composition and relative abundance of vaginal microbiota has been determined by high-throughput 16s rRNA sequencing, characterizing five microbial community state types (CST) in asymptomatic women (Ravel et al., 2011). Four of them (CST-I, II, III, V) were dominated by *Lactobacillus* species, while CST-IV was heterogenous and polymicrobial, characterized by lower level of *Lactobacillus* and higher level of anaerobic bacteria including *Gardnerella*, *Atopobium*, *Mobiluncus*, *Prevotella*, *Streptococcus*, *Mycoplasma*, and *Ureaplasma* (Ravel et al., 2011). At present, over 140 *Lactobacillus* species have been identified, but the only species that normally dominate the vaginal microbiota are *L. crispatus*, *Lactobacillus gasseri*, *Lactobacillus jensenii*, and *L. iners* (Smith and Ravel, 2017). They are considered as keystones of vaginal health, as they can produce lactic acid, hydrogen peroxide, and bacteriocins, maintaining acidic environment and preventing pathogen growth (Gupta et al., 2019); adhere to epithelium, repelling other bacteria adhesion (Torcia, 2019); and regulate immune and inflammatory response, enhancing the resistance of vagina to diseases (Aldunate et al., 2015). Thus, *Lactobacillus* dominance is generally considered as a hallmark of healthy vagina (Ma et al., 2012).

It is generally known that vaginal microbiota disturbance is highly related to various gynecological diseases, especially BV, which is characterized by the alteration of vaginal microbiome from *Lactobacillus* dominance to anaerobic and facultative bacteria (*Gardnerella*, *Atopobium*, *Prevotella*, *Megasphaera*, *Leptotrichia*, *Sneathia*, etc.) dominance (Ling et al., 2010; Srinivasan et al., 2012; Nasioudis et al., 2017). BV has been shown to be associated with various other reproductive tract disorders, including infertility, preterm, cervical cancer, and HIV acquisition (Leitich and Kiss, 2007; van Oostrum et al., 2013; Torcia, 2019). It is also reported that many sextually transmitted infections (STI), such as infections of *N. gonorrhoeae* and *C. trachomatis*, are facilitated by vaginal microbiota dysbiosis and more prevalent in BV-positive women (Lewis et al., 2017). In addition, as the microbiota research progresses intensively, a growing number of studies have linked vaginal microbiota dysbiosis to various gynecological non-infectious diseases, among which Liu et al. found that compared with healthy people, IUA patients had lower percentage of *Lactobacillus*, and higher percentage of *Gardnerella* and *Prevotella* (Liu et al., 2019); Hong et al. found that patients with PCOS had lower *Lactobacillus* and higher *Mycoplasma* and *Prevotella* than controls (Hong et al., 2020b); and Chen et al. found that *Lactobacillus* were less abundant, while *L. iners* were more abundant in patients with uterine fibroid than individuals without (Chen et al., 2017).

Therefore, as the balanced vaginal microbiota plays a significant role in female health, interventions aimed at restoring the healthy microbiota composition can be a good and reasonable therapy for gynecological diseases.

## Role of Microbiota Dysbiosis in Infectious Diseases and Interventions

**FIGURE 1:**
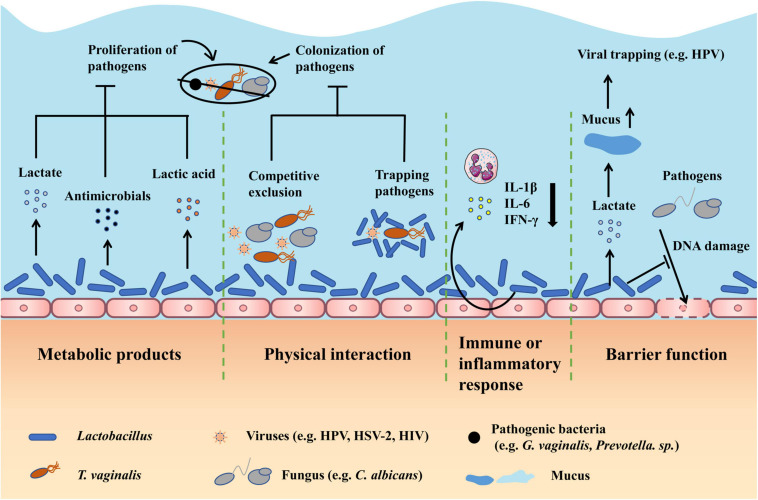
Therapy effects of restoring *Lactobacillus*-dominated vaginal microbiota in infectious diseases. First, *Lactobacillus* can produce metabolites including lactate, antimicrobials, and lactic acid to inhibit the proliferation of pathogens. Second, *Lactobacillus* can competitively exclude pathogens from adhering to epithelium and trap pathogens by direct physical contact to prevent the colonization of pathogens. Third, *Lactobacillus* can regulate the immune or inflammatory response, particularly relieving the inflammation by decreasing cytokines like IL-1β. Fourth, *Lactobacillus* can improve the barrier function by producing lactate, which can increase the mucus viscosity to facilitate viral trapping, and inhibiting pathogens from damaging the DNA of epithelial cells. DNA, deoxyribonucleic acid.

Until now, infection is one of the leading causes of gynecological disease, which is usually characterized by vaginal dysbiosis (van de Wijgert and Jespers, 2017). Below, we introduce the common infectious diseases, including common vaginitis, viral infections, and other infections, and discuss the therapy effects of restoring *Lactobacillus*-dominated vaginal microbiota by antibiotic and probiotic interventions (Figure 1).

### Microbiota Dysbiosis and Interventions in Common Vaginitis

#### Microbiota Dysbiosis in BV and Interventions

Bacterial vaginitis is the most common lower genital tract disease among fertile women and can predispose women to various STI and adverse birth outcome (Lewis et al., 2017). It mainly manifests as mucosal inflammation including abnormal vaginal discharge (increased, yellowish, and fishy odor) and sensation of itching and burning (Onderdonk et al., 2016). Currently, the routine treatment is oral and intravaginal antibiotics, usually clindamycin and metronidazole (Faught and Reyes, 2019). However, long-term use of antibiotics is likely to develop antimicrobial resistance and cause recurrent infections (Lev-Sagie et al., 2019).

Studies showed that BV is caused by replacement of *Lactobacillus* dominance by multiplication of over 10 anaerobic bacteria, such as *Gardnerella*, *Atopobium*, *Prevotella*, *Megasphaera*, *Leptotrichia*, and *Sneathia*, and probiotics based on *Lactobacillus* that used to regulate microbiota have been revealed beneficial in treating BV (Nasioudis et al., 2017; Wang et al., 2019). *In vitro* and clinical studies showed that *Lactobacillus* could reduce the pathogen colonization by preventing the pathogen adhering to epithelium (Ma et al., 2019), inhibit the pathogen growth by producing bacteriocins (Ma et al., 2019), maintain the acidic environment by generating lactic acid (Kim and Park, 2017), and relieve inflammatory response, in particular significantly reduce IL-1β and IL-6 cytokines (Hemalatha et al., 2012). Thus, antibiotics with probiotics can effectively cure BV by correcting the vaginal microbiota and improving the vaginal environment.

#### Microbiota Dysbiosis in VVC and Interventions

Vulvovaginal candidiasis is the most common vaginal fungal infection and typically manifests as mucosal inflammation, including cheese-like vaginal discharge, and vulvovaginal burning, itching, and redness (Sobel, 1992). The standard treatment for VVC is antifungal agents, including oral or intravaginal azole or triazole drugs, which can achieve over 80% cure rate (Nurbhai et al., 2007; Sobel and Sobel, 2018). However, the concomitant side effects (diarrhea, abnormal urination, and vaginal burning, itching, and irritation), drug resistance, and high recurrence rate hinder the recovery and pose a threat to health (Xie et al., 2017).

Studies showed that VVC primarily occurred during vaginal dysbiosis and immune deficiency, and was caused by overgrowth of *C. albicans*, which could cause epithelium destruction by destroying the intercellular linkage and intracellular mitochondrial structure, and elicit inflammation, in particular produce IL-6 and IL-8 cytokines (Niu et al., 2017; Li et al., 2019). As protective *Lactobacillus* can regulate the host immune response, inhibit the proliferation of *C. albicans* by producing metabolites such as lactate, and prevent the colonization of *C. albicans*, therapies aimed at adjusting microbiota can help in VVC recovery (Bradford and Ravel, 2017). *In vivo* study on VVC showed that *Lactobacillus* could regulate the immune response by decreasing T-helper 1 (Th1) cell/Th2 cell ratio and inhibiting the release of proinflammatory cytokines such as interleukin 17 (IL17) and interferon-γ (IFN-γ) (Li et al., 2019). Another *in vitro* study investigated the ability of *L. crispatus* to inhibit *C. albicans* infecting vaginal epithelial cells VK2/E6E7, and found that *L. crispatus* could significantly reduce the adherence of *C. albicans* to VK2/E6E7 cells (Niu et al., 2017). Besides, many studies suggested that *Lactobacillus* could exert direct antifungal effects by releasing antimicrobials, improving the epithelial barrier by decreasing DNA damage of epithelial cells, and improving the microbiota to prevent *C. albicans* overgrowth and VVC recurrence (Mc and Rosenstein, 2000; Reid et al., 2003; Strus et al., 2006; Yeh et al., 2007). Consequently, antibiotics with probiotics can treat VVC and prevent its onset by improving microbial, inflammatory, and epithelial status.

#### Microbiota Dysbiosis in Trichomonal Vaginitis and Interventions

Trichomonal vaginitis, which manifests as painful and itching coitus, frothy discharge, and vaginal or cervical bleeding, is the most common STI worldwide and closely linked to PID and infertility (Pastorek et al., 1996; Petrin et al., 1998; Harp and Chowdhury, 2011; Edwards et al., 2016). Currently, the standard treatment for Trichomonal vaginitis is metronidazole or tinidazole, which not only are quite effective but also develop drug resistance (Muzny and Schwebke, 2013; Phukan et al., 2018).

The etiology of trichomonal vaginitis is *T. vaginalis*, which is a flagellated parasite of human genital tract that can cause severe damage to epithelial cells by mediating the lysis of epithelial cells, and elicit the inflammatory response, involving recruitment of neutrophils to infected tissues (Fichorova et al., 2006; Edwards et al., 2016). Studies showed that BV, especially with lack of *Lactobacillus*, could protect *T. vaginalis* from nucleic acid degradation through the production of polyamines and form biofilms to reduce drug sensitivity, thus facilitating *T. vaginalis* infection and increasing drug resistance (Figueroa-Angulo et al., 2012; Balkus et al., 2014; Jung et al., 2017). Therefore, it is suggested that restoring the vaginal microbiota can improve the anti-trichomoniasis treatment effect. A randomized clinical study showed that patients taking probiotics in addition to metronidazole showed earlier clinical resolution compared with the patients taking placebo. Besides, reduced leukocyte/epithelial cell ratio, decreased pH, and increased redox potential of the vaginal fluid were detected in the probiotic group, underlining the beneficial mechanisms of *Lactobacillus* to relieve inflammation, inhibit *T. vaginalis* growth, and damage T. vaginalis DNA, respectively (Sgibnev and Kremleva, 2020). Another *in vitro* study showed that the aggregation-promoting factor (APF)-2 of *L. gasseri* could significantly inhibit the adhering of *T. vaginalis* to human vaginal ectocervical cells (Phukan et al., 2018). Thus, antibiotics with probiotics can prevent and treat *T. vaginalis* infection via adjusting vaginal microbiota, inhibiting *T. vaginalis* growth, relieving inflammation, and preventing *T. vaginalis* colonization.

#### Microbiota Dysbiosis in AV and Interventions

Atrophic vaginitis, caused by the reduction of estrogen and local immunity after menopause, is prevalent among postmenopausal women and is characterized by vulvovaginal dryness, dyspareunia, abnormal vaginal discharge, etc. (Stika, 2010). At present, the treatment principles of AV are giving estrogen to improve the vaginal immunity, and antibiotics (norfloxacin) to inhibit the pathogen growth (Paladine and Desai, 2018).

Estrogen deficiency in menopause leads to vaginal atrophy, resulting in reduced epithelial barrier function and facilitation of pathogen colonization (Jaisamrarn et al., 2013). Brotman et al. (2014) showed that women with AV had lower level of *Lactobacillus* and increased bacterial diversity, involving *Anaerococcus*, *Peptoniphilus*, *Prevotella*, and *Streptococcus*. Since *Lactobacillus* has been shown to improve the overall vaginal environment such as the vaginal immunity and epithelial barrier, probiotics have been used in combination with estrogen to treat AV. A randomized clinical study showed that long-term use of this combination was sufficient to improve the related clinical parameters, maintain the improved maturation of vaginal epithelium, and prevent symptomatic AV relapse (Jaisamrarn et al., 2013). Besides, it is suggested that *Lactobacillus*-dominated vaginal microbiota is significant in protecting postmenopausal women from AV and is considered to be a marker of successful AV treatment (Shen et al., 2016). Thus, antibiotics and probiotics can be used in combination with estrogen to prevent and treat AV by improving the vaginal microbiota and enhancing the epithelial barrier function and the overall vaginal environment.

### Microbiota Dysbiosis and Interventions in Viral Infection

Genital HPV, especially HPV-16 and 18 strains, are common sextually transmitted viruses and major causes of cervical cancer (Stanley, 2010). Though over 50% of HPV infections are cleared within a half year, persistent infections can cause symptoms including abnormal vaginal bleeding such as sextual intercourse bleeding, and abnormal vaginal discharge (Mitra et al., 2016; Kashyap et al., 2018). Conventional treatments for cervical cancer include surgery, chemotherapy, and radiotherapy, but they cannot prevent recurrence and have various side effects, such as menstrual change and vaginal pain (Waggoner, 2003).

Studies showed that vaginal microbiota disturbance with reduced *Lactobacillus* and increased microbial diversity was closely linked to HPV infection pathogenesis (Mitra et al., 2016). A cohort study of 32 sextually active American women showed that women with high level of *Atopobium*, *Prevotella*, and *Gardnerella* were most likely to be infected with HPV and had the slowest viral clearance (Brotman et al., 2014). Besides, CST-IV bacteria were shown to increase the severity of cervical lesions, promote the neoplastic progression by producing nitrosamines and ROS to induce DNA damage, and facilitate HPV infection by damaging the barrier and eliciting chronic inflammation (Mhatre et al., 2012; Borgdorff et al., 2016; Piyathilake et al., 2016). Thus, improving the microbiota composition is a feasible approach to prevent and treat HPV infection. A semi-randomized study of 54 HPV infected women showed that women treated with oral *Lactobacillus caseii* had greater clearance of HPV infection and cervical lesions than untreated patients (Verhoeven et al., 2013). Mitra et al. reviewed the currently accepted mechanisms of *Lactobacillus*-mediated protection to cervical health and showed that low vaginal pH (which could decrease around 10% risk of HPV positivity), lactate (which could increase the vaginal mucus viscosity and enhance the viral trapping), and bacteriocins (which could directly interfere the pathogen growth) were significant protective principles, and improvement in local immunity and inflammation was also suggested (Mitra et al., 2016). In addition, Palma et al. (2018) explored the effects of long- and short-term probiotic implementation in clearing HPV infection, and they found that patients with long-term probiotic treatment had significantly higher viral clearance rate, suggesting that long-term healthy vaginal microbiota was required to exert the optimal treatment effect. Therefore, antibiotics and probiotics can prevent and aid treatment of HPV infection by improving the microbial balance.

Other common viral agents that infect the reproductive tract are HSV-2 and HIV, which can cause genital ulcers and AIDS, respectively. Since these viruses cannot be completely eliminated by traditional antiviral therapy, prevention is particularly important for health maintenance. Studies showed that vaginal dysbiosis, especially BV, and lack of *Lactobacillus* could facilitate HSV-2 infection, and a protective vaginal microbiota could prevent and counteract HSV-2 infection by inhibiting HSV-2 replication, producing antimicrobials, and trapping HSV-2 particles (Cherpes et al., 2003; Evans et al., 2003; Conti et al., 2009; Mousavi et al., 2018; Torcia, 2019). As for HIV, many studies showed that women with CST-IV bacteria had a higher HIV infection rate than women with CST-I bacteria, and a *Lactobacillus*-dominated vaginal microbiota could protect the vagina from HIV infection by maintaining acidic environment, producing lactic acid, and reducing the viability of HIV particles (Gosmann et al., 2017; Nahui Palomino et al., 2017). Therefore, antibiotics with probiotics can prevent HIV and HSV-2 infection by correcting the microbial disturbance and creating an unfavorable vaginal environment for viruses.

### Microbiota Dysbiosis and Interventions in Other Infections

*Neisseria gonorrhoeae*, the causative agent of gonorrhea, is continuously developing resistance to antimicrobial treatment (ceftriaxone and azithromycin) and impedes the recovery (Morgan and Decker, 2016; Unemo et al., 2019). An *in vitro* study showed that *Lactobacillus* could significantly reduce *Neisseria gonococcus* viability by creating acidic environment, producing bacteriocins, releasing biosurfactants, and co-aggregating with gonococci, and reduce gonococci adhering to epithelial cells (Foschi et al., 2017). Thus, probiotics can be an adjuvant therapy of antibiotics to treat *N. gonorrhoeae* infection by improving the vaginal microbiota.

*Mycoplasma genitalium* is a sextually transmitted pathogen that can lead to PID and cervicitis (Cazanave et al., 2012; Onderdonk et al., 2016), and its first line treatment with antibiotics (doxycycline and azithromycin) is compromised by drug resistance (Pinto-Sander and Soni, 2019). Studies showed that BV could favor *M. genitalium* infection (Brotman et al., 2010; Molenaar et al., 2018), and a protective vaginal microbiota dominated by *Lactobacillus* could counteract the infection by producing antimicrobials and maintaining acidic environment (Molenaar et al., 2018). Therefore, probiotics with antibiotics can prevent and treat *M. genitalium* infection by adjusting the microbial structure and improving the vaginal environment.

*Chlamydia trachomatis* is a common cause of PID (Haggerty et al., 2010; Mestrovic and Ljubin-Sternak, 2018), and its treatment with azithromycin or doxycycline also faces the challenge of antimicrobial resistance (O’Connell and Ferone, 2016; Zhang et al., 2017; Mestrovic and Ljubin-Sternak, 2018). *In vitro* studies showed that *Lactobacillus* could prevent *Chlamydia* colonization by maintaining acidic environment and consuming glucose (Nardini et al., 2016), inhibit *Chlamydia* multiplication at all infection stages, and inhibit the chronic infection by preventing the development of persistent *trachomatis* forms (Mastromarino et al., 2014). Therefore, antibiotics with probiotics can exert a conducive treatment effect on *C. trachomatis* infection by improving the vaginal microbiota.

## Role of Microbiota Dysbiosis in Non-Infectious Diseases and Interventions

**FIGURE 2:**
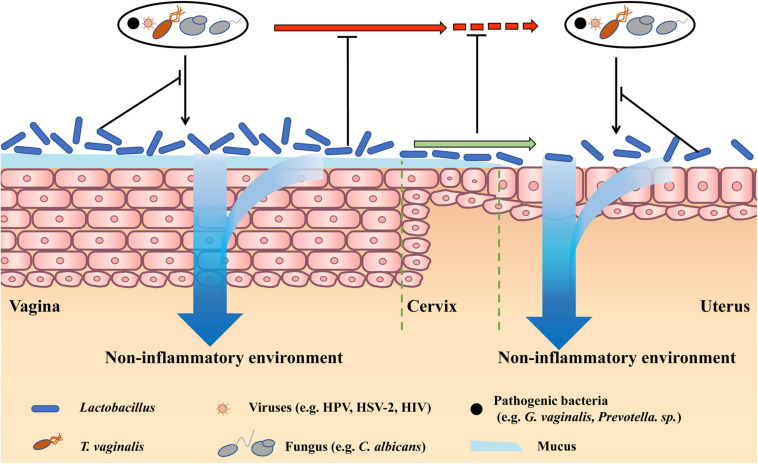
Therapy effects of restoring *Lactobacillus*-dominated vaginal microbiota in non-infectious diseases. *Lactobacillus*-dominated vaginal microbiota cannot only benefit vaginal health by preventing vaginal infections and creating a non-inflammatory environment but also prevent pathogens in the vagina moving to the cervix and uterus. Besides, vaginal *Lactobacillus* can move to the upper reproductive tract, preventing pathogens infecting the cervix and uterus, and creating a non-inflammatory cervical and uterine environment.

The role of microbiota in non-infectious gynecological diseases was long underestimated, until recently when mounting evidence showed its significance. Herein, we introduce the common non-infectious diseases that were caused by physical injury, fertility problems, and endocrine disorders, and discuss the treatment effects of restoring *Lactobacillus*-dominated vaginal microbiota by antibiotic and probiotic interventions (Figure 2).

### Microbiota Dysbiosis and Interventions in Diseases Caused by Physical Injury

Induced abortion is a fairly common gynecological operation worldwide, and whether it is drug-induced or surgery-induced, it can cause a great damage to the female reproductive tract and predispose to serious complications such as incomplete abortion, heavy bleeding, infection, scarring of endometrium, adhesions of the uterine cavity and cervix, and endometriosis. Among these, upper genital tract infection is the most concerned and common clinical problem, which can further cause endometritis, salpingitis, and infertility (Mary and Mahmood, 2010; Carlsson et al., 2018).

Studies showed that postabortal infection was usually caused by pathogens from the lower genital tract, such as *Chlamydia*, *N. gonorrhoeae*, *Mycoplasma*, and BV-related bacteria, moving through the cervix to the uterus and even to fallopian tubes, causing infections and inflammation of the entire reproductive system (Bjartling et al., 2010). In this regard, World Health Organization (WHO) recommended the use of antibiotic prophylaxis to prevent the abortion-induced infection, and a meta-analysis of randomized clinical studies showed that perioperative antibiotics (metronidazole, nitroimidazoles, etc.) could effectively reduce the risk of postabortal infection by 50% (Sawaya et al., 1996; Carlsson et al., 2018). However, if the homeostasis of the uterus is not fully restored and is still conducive to microbial disorders, antibiotics cannot protect the women from being reinfected (Low et al., 2012). Therefore, as probiotics have a great potential in restoring the normal vaginal microbiota and improving the uterine environment, perioperative use of antibiotics and probiotics can prevent the postabortal infection.

Intrauterine adhesions is a condition in which the scar tissues build up inside the uterine cavity, which in many cases will cause adhesion of the opposing endometrium (Chi et al., 2018). Though the adhesions can be removed through the transcervical resection, the postoperative recurrence is high (Pabuccu et al., 2008). A clinical study showed that IUA patients had disturbed vaginal microbiota, characterized by obviously reduced probiotic *Lactobacillus*, and increased pathogenic *Gardnerella* and *Prevotella*, which may promote the uterine pathology and cause recurrence (Liu et al., 2019). Therefore, antibiotics and/or probiotics can promote the recovery and reduce the recurrence of IUA by correcting the vaginal microbiota.

### Microbiota Dysbiosis and Interventions in Diseases Caused by Fertility Problems

Miscarriage, defined as a spontaneous pregnancy loss before the expected point, is the most prevalent pregnancy complication (Smith et al., 2019). The etiology is multifactorial and usually secondary to other disorders, such as uterine malformation, infections, chromosomal abnormalities, and hormone deficiency (progestogen) (Haas et al., 2019). In this regard, the preventive and curative measures are always aimed at addressing the primary causes (Haas et al., 2019).

Previous studies showed that pregnant women with BV had around two-fold higher risk of miscarriage than those without BV, which was thought to be caused by ascending of the BV-related bacteria from the vagina to the uterus, leading to endometritis, deciduitis, chorioamnionitis, and amniotic fluid infection (Ralph et al., 1999; Goldenberg et al., 2000). Studies exploring the specific mechanisms of BV favoring miscarriage showed that BV-related bacteria could produce lytic enzymes (proteases, phospholipases, etc.) to cause lysis of the fetal membranes, and induce the formation of prostaglandin, which could promote the uterine muscle contraction, decrease the cervical resistance, and induce the release of metalloproteinases (MMPs) to degrade the chorioamniotic membranes (Isik et al., 2016). Besides, the level of inflammatory cytokines IL-6 and IL-8 was also elevated in the amniotic fluid of pregnant women with BV (Keelan et al., 1997). A randomized controlled trial showed that pregnant women treated with clindamycin for BV were five times less likely to miscarry than those given placebo (Ugwumadu et al., 2003). This suggests that early screening and treatment of BV can prevent the infection-induced miscarriage. In addition, a clinical study suggested that hydrogen peroxide producing *Lactobacillus* in the vagina could not only benefit the vaginal health by improving the microbial and inflammatory status, but also create a favorable uterine environment for implantation and placentation (Eckert et al., 2003). Thus, antibiotics and probiotics could prevent and treat infection-induced miscarriage by restoring the vaginal microbiota and improving the intrauterine environment.

Plenty of evidence showed that BV could also increase the risk of preterm and infertility, mainly via facilitating STI and eliciting the intrauterine inflammation (Peelen et al., 2019; Hong et al., 2020a). Recent studies showed that *Lactobacillus*-dominated vaginal microbiota was negatively associated with preterm and infertility, and could prevent women from adverse fertility outcome by modulating vaginal microbiota and inflammatory cytokines IL-4 and IL-10 (Vitali et al., 2012; Hong et al., 2020a). Therefore, antibiotics and probiotics can prevent the preterm and infertility by improving the vaginal and uterine microbiota and eubiosis.

### Microbiota Dysbiosis and Interventions in Diseases Caused by Endocrine Disorders

Polycystic ovarian syndrome is one of the most prevalent endocrine disorders in reproductive women, which usually manifests as menstrual disorder, hirsutism, and infertility (Hong et al., 2020b). Though current treatments are various, such as oral contraceptives to inhibit the maturation of ovarian follicles as a long-term PCOS management, and ovulation induction for PCOS patients with fertility requirement, they cannot cure it basically and lifestyle modification (e.g., loss weight) is still the first-line and mainstream treatment (Jin and Xie, 2018).

A case–control study of 39 PCOS patients and 40 healthy people showed that the vaginal microbiota of PCOS patients was significantly different from that of healthy people, characterized by increased diversity and increased relative abundance of *Mycoplasma* and *Prevotella*, and decreased relative abundance of *L. crispatus* (Hong et al., 2020b). This suggests that vaginal microbiota dysbiosis may participate or contribute to the PCOS pathology, and therapies targeted at improving the vaginal microbiota are promising. A systemic review involving 855 PCOS patients investigated the effects of probiotics (*Lactobacillus*) in treating PCOS, and the results showed that probiotic supplementation in PCOS women significantly improved their hormonal index by reducing free androgen index (FAI) and increasing sex hormone binding globulin (SHBG), and their inflammatory index by increasing plasma nitric oxide (NO) and reducing blood malondialdehyde (MDA) (Shamasbi et al., 2020). Besides, the authors also observed that patients given probiotics had increased total glutathione (GSH) and total antioxidant capacity (TAC) levels, and reduced testosterone, dehydroepiandrosterone sulfate (DHEAS, hormonal index), high sensitive C reactive protein (hsCRP, inflammatory index), and hirsutism score compared to those given placebo. As antibiotics and probiotics are aimed at restoring the normal vaginal microbiota and improving the environment of the reproductive system and beyond, they can be used to treat the PCOS symptoms and promote the recovery by improving the hormonal and inflammatory levels.

Recently, microbiota disturbance has also been implicated in the uterine fibroid (benign tumors in the uterus) and menstrual disorders (e.g., menorrhagia and dysmenorrhea), characterized by increased relative abundance of vaginal *L. iners*, and increased relative abundance of uterine *Gardnerella*, *Prevotella*, *Sneathia*, and *Veillonella*, respectively (Chen et al., 2017; Pelzer et al., 2018). Thus, antibiotics and/or probiotics are also promising in treating uterine fibroid and menstrual disorders.

## Frontiers and Conclusion in Treating Vaginal Dysbacteriosis

**FIGURE 3:**
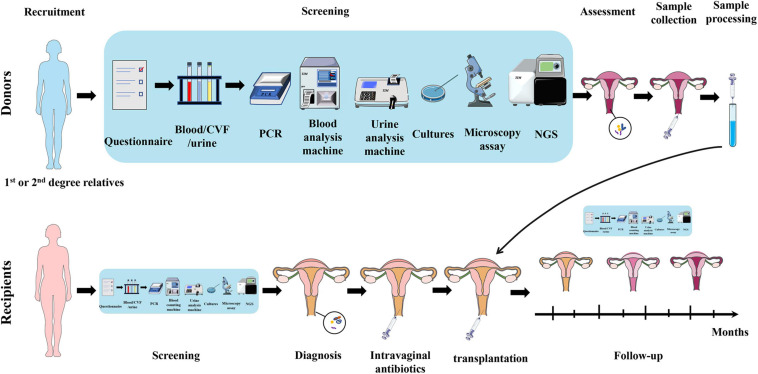
Schematic diagram of VMT operation. As for donors, first, donors are recruited, preferably from the recipients’ first and second relatives. Second, collect blood/CVF/urine samples of donors and conduct a series of screening by questionnaire, PCR, blood, and urine analysis machines, cultures, microscopy, and NGS. Third, collect CVF sample from qualified healthy donor and process it. It is then transplanted into the recipient’s vagina. As for recipients, first, recipients undergo the same screening process for diagnosis and basic health assessment. Second, recipients are given intravaginal antibiotics to prepare for transplantation. Third, transplant the prepared CVF solution from healthy donor into the recipient’s vagina. Fourth, follow-up studies are carried out on recipients to assess the treatment outcome and adverse effects. VMT, vaginal microbiota transplantation; CVF, cervicovaginal fluid; PCR, polymerase chain reaction; NGS, next-generation sequencing.

Inspired by the similarity of the intestinal and vaginal microbiota, and the success of the fecal microbiota transplantation (FMT), VMT has also been proposed for the treatment of vaginal dysbacteriosis, which involves transplanting the entire vaginal microbiota of a healthy donor into the vagina of the patient to restore the overall diversity, stability, and normal composition of the microbiota (DeLong et al., 2019). The procedures of VMT are shown in Figure 3. The research of our group in 2017 showed that transplantation of healthy rat vaginal microbiota into the vagina of BV model rats restored the morphology of uterine tissue and reduced the serum inflammatory factors such as IL-6, IL-8, and TNF-α, showing obvious recovery effects on vaginal infections caused by dysregulation of vaginal microbiota. In 2019, the clinical study conducted by Lev-Sagie et al. (2019) further showed that VMT had a great effect on long-term recovery from recurrent, antibiotic unresponsive, and refractory BV. In this study, four of the five BV patients treated with VMT recovered effectively after 5–21 months of VMT treatment, showing significant improvements in symptoms, negative Amsel criteria, and *Lactobacillus*-dominated vaginal fluid under the microscopy, with a cure rate up to 80% and no observed adverse effect. Besides, the authors also found that patients with long-term resolution of BV had a dramatic change in microbial composition in the first month after VMT, which was dominated by increase of *Lactobacillus* and decrease of *Bifidobacterium* (closely related to *Gardnerella*), accompanied with reduced *Fannyhessea* and *Prevotella*. In 2021, our group verified the feasibility of VMT in animal models and explored the specific mechanisms (Chen et al., 2021). The results showed that vaginal secretions from healthy rats can be used to treat vaginal microbiota imbalance and prevent the recurrence in rats, which specifically manifested as the decrease of inflammatory cells, pro-inflammatory cytokines, and apoptotic factors in the uterine wall and the restoration of the diversity of vaginal microbiota.

Preliminary studies of VMT have demonstrated the feasibility of VMT to treat BV, showing favorable therapeutic effects. Compared to other treatments for BV, VMT can completely restore the vaginal microbiota to a healthy state, thus showing better curative effects than conventional antibiotics and probiotics, while addressing the drug resistance, recurrence, and side effects associated with antibiotic treatment. Considering that besides BV, vaginal microbiota dysbiosis is also comprehensively involved in the progression of other gynecological diseases, and improving vaginal microbiota by antibiotics and probiotics shows good therapy effects, restoration of vaginal microbiota by VMT may also have favorable therapeutic effects in the treatment of various gynecological infectious and non-infectious diseases.

However, the clinical implementation of VMT still faces many problems, such as insufficient VMT clinical trial (only one research with five subjects), lacking standard protocol, transmission of unidentifiable and antimicrobial-resistance pathogens, unintended pregnancy, immune rejection, and unclear long-term effects. Therefore, the improvement of the VMT requires multi-disciplinary cooperation. Relevant personnel should formulate VMT screening guidelines as soon as possible, continue to explore the application potential of VMT in the treatment of BV and other gynecological diseases, develop a safe and effective new treatment regimen, and develop safety evaluation criteria. We have reasons to believe that safe, standard, and efficient VMT will bring new hope to patients with gynecological diseases and have a good prospect of application.

## Author Contributions

TC and ZL provided ideas of this review and designed its framework. YH conducted the research and wrote the manuscript. All authors edited the manuscript. All authors read and approved the final manuscript.

## Conflict of Interest

The authors declare that the research was conducted in the absence of any commercial or financial relationships that could be construed as a potential conflict of interest.
